# Regulation of Human γδ T Cells by BTN3A1 Protein Stability and ATP-Binding Cassette Transporters

**DOI:** 10.3389/fimmu.2018.00662

**Published:** 2018-04-04

**Authors:** David A. Rhodes, Hung-Chang Chen, James C. Williamson, Alfred Hill, Jack Yuan, Sam Smith, Harriet Rhodes, John Trowsdale, Paul J. Lehner, Thomas Herrmann, Matthias Eberl

**Affiliations:** ^1^Department of Pathology, University of Cambridge, Cambridge, United Kingdom; ^2^Division of Infection and Immunity, School of Medicine, Cardiff University, Cardiff, United Kingdom; ^3^Cambridge Institute for Medical Research, University of Cambridge School of Clinical Medicine, Cambridge, United Kingdom; ^4^Institut für Virologie und Immunbiologie, Julius-Maximilians-Universität Würzburg, Würzburg, Germany; ^5^Systems Immunity Research Institute, Cardiff University, Cardiff, United Kingdom

**Keywords:** butyrophilins, T cells, phosphoantigens, mevalonate pathway, ABCG2, NRF2

## Abstract

Activation of human Vγ9/Vδ2 T cells by “phosphoantigens” (pAg), the microbial metabolite (*E*)-4-hydroxy-3-methyl-but-2-enyl pyrophosphate (HMB-PP) and the endogenous isoprenoid intermediate isopentenyl pyrophosphate, requires expression of butyrophilin BTN3A molecules by presenting cells. However, the precise mechanism of activation of Vγ9/Vδ2 T cells by BTN3A molecules remains elusive. It is not clear what conformation of the three BTN3A isoforms transmits activation signals nor how externally delivered pAg accesses the cytosolic B30.2 domain of BTN3A1. To approach these problems, we studied two HLA haplo-identical HeLa cell lines, termed HeLa-L and HeLa-M, which showed marked differences in pAg-dependent stimulation of Vγ9/Vδ2 T cells. Levels of IFN-γ secretion by Vγ9/Vδ2 T cells were profoundly increased by pAg loading, or by binding of the pan-BTN3A specific agonist antibody CD277 20.1, in HeLa-M compared to HeLa-L cells. IL-2 production from a murine hybridoma T cell line expressing human Vγ9/Vδ2 T cell receptor (TCR) transgenes confirmed that the differential responsiveness to HeLa-L and HeLa-M was TCR dependent. By tissue typing, both HeLa lines were shown to be genetically identical and full-length transcripts of the three BTN3A isoforms were detected in equal abundance with no sequence variation. Expression of BTN3A and interacting molecules, such as periplakin or RhoB, did not account for the functional variation between HeLa-L and HeLa-M cells. Instead, the data implicate a checkpoint controlling BTN3A1 stability and protein trafficking, acting at an early time point in its maturation. In addition, plasma membrane profiling was used to identify proteins upregulated in HMB-PP-treated HeLa-M. ABCG2, a member of the ATP-binding cassette (ABC) transporter family was the most significant candidate, which crucially showed reduced expression in HeLa-L. Expression of a subset of ABC transporters, including ABCA1 and ABCG1, correlated with efficiency of T cell activation by cytokine secretion, although direct evidence of a functional role was not obtained by knockdown experiments. Our findings indicate a link between members of the ABC protein superfamily and the BTN3A-dependent activation of γδ T cells by endogenous and exogenous pAg.

## Introduction

Gammadelta (γδ) T cells are a lineage of innate-like “unconventional” T lymphocytes with potent cytotoxic, pro-inflammatory, and regulatory properties. They express, as a defining feature, a T cell receptor (TCR) composed of a γ and δ chain heterodimer, both products of V(D)J recombination, which distinguishes them from conventional αβ T cells (1).

Although often restricted to distinct anatomical locations including skin and intestinal epithelium, both major sites of host interaction with the microbiota and of pathogen entry, the role of γδ T cells in tissue immune surveillance is not fully understood (2, 3). Butyrophilins (BTNs) appear to be pivotal in the maintenance of immune homeostasis in tissue epithelium by controlling the activation of γδ T cells (4). The observed homology to co-receptors, such as CD80, CD86, and PD-L1 (5), initially suggested the possibility of ligands for BTN molecules expressed on the surface of T cells, although identification of such receptors has remained elusive so far (6). Some data are consistent with a direct interaction with the γδ TCR, although this remains to be confirmed (7–9).

Our work aims to understand the function of BTNs in the regulation of γδ T cells and how their dysregulation may contribute to disease. We study the human BTN3A proteins and their role in the activation of Vγ9/Vδ2 T cells, a prominent γδ T cell lineage in human blood and tissues (10). A mandatory role for paired BTN3A molecules in controlling Vγ9/Vδ2 T cell activation has been shown recently (11), and other BTN and BTN-like molecules both in mouse and in humans may similarly control defined γδ T cell subsets. For example, Skint1 determines the Vγ5/Vδ1 dendritic epidermal T cell lineage in murine skin (12, 13), Btnl1 controls the Vγ7+ mouse enterocyte γδ T cell compartment, and BTNL3 and BTNL8 control human colonic Vγ4+ γδ T cells (4). Therefore, this work is likely to be relevant to other systems of γδ T cell selection, homeostasis, and antigen-dependent activation.

The Vγ9/Vδ2 lineage of γδ T cells is known to be activated by exposure to specific “phosphoantigens” (pAg), isopentenyl pyrophosphate (IPP) and (*E*)-4-hydroxy-3-methyl-but-2-enyl pyrophosphate (HMB-PP). IPP is a ubiquitous metabolite in all living cells and the common end product both of the classical mevalonate (also called the HMG-CoA reductase) pathway in eukaryotes and some bacteria and of the alternative non-mevalonate pathway in Gram-negative bacteria, mycobacteria, and malaria parasites. The microbial metabolite HMB-PP is an intermediate of the non-mevalonate pathway, showing 4log10 greater activity over IPP (14, 15). While IPP has a lower bioactivity on Vγ9/Vδ2 T cells in cell culture and lower affinity for the B30.2 domain of BTN3A1 in binding assays compared to HMB-PP, IPP may still play a physiological role *in vivo*. This may be the case where intracellular IPP levels are elevated as a result of dysregulation of the mevalonate pathway, for instance upon treatment of target cells with aminobisphosphonate drugs such as zoledronate, by infection or cellular transformation (16, 17).

In addition to cytokine production and cytotoxicity, Vγ9/Vδ2 cells promote conventional peptide antigen presentation *via* MHC-I- and MHC-II-dependent mechanisms (18–20) and are being investigated as potential tools in cancer immunotherapy (21–26). Further, while γδ T cells generally are linked to inflammatory and autoimmune responses (27, 28), the contribution of the Vγ9/Vδ2 lineage to autoimmune pathology has not been addressed. In these contexts, understanding the molecular basis of their activation by BTN3A-dependent pAg presentation should facilitate their therapeutic manipulation (29).

In initial screens, we identified two HeLa cell lines which differed markedly in their ability to elicit cytokine from cocultured γδ T cells. We have used this model to investigate the function of BTN3A molecules in this process and to identify novel components. The data indicate that environmental exposure, such as treatment of cells with the cytotoxic anticancer drug doxorubicin (DOX), may influence γδ T cell activation by regulation of BTN3A protein stability or trafficking and expression of ATP-binding cassette (ABC) transporters *via* the KEAP1/NRF2 stress response pathway.

## Materials and Methods

### Expression Constructs

Primer sequences are listed in Table S1 in Supplementary Material. DNA encoding specific shRNA oligos directed to BTN3A isoforms, periplakin, and ABCG2 were cloned into pHR-SIREN/puro using *Bam*HI/*Eco*RI as described previously (30). Virus particles were produced by co-transfection of 293T cells with pCMV8.91 (gag-pol) and pMDG (VSV-G env) plasmids. Supernatant was harvested after 48 h, filtered, and used to transduce HeLa cells. Clones were selected by puromycin (1 µg/ml) for 24 h. shRNA oligonucleotide sequence targeting all BTN3A isoforms was: shBTN3A.CGTGTATGCAGATGGAAAG. For re-expression, HeLa shRNA^BTN3A^ cells were co-transduced with lentivirus carrying variant BTN3A1 sequences. Green fluorescent protein (GFP) positive cells were sorted using BD FACSAria cell sorter.

### Cell Lines and DNA Constructs

HeLa-M (a gift from Dick van den Boomen CIMR, Cambridge) and HeLa-L (from Will McEwan, MRC-LMB, Cambridge) cervical carcinoma cells were grown in standard tissue culture conditions using RPMI-1640 medium plus 10% FCS, penicillin/streptomycin (100 U/ml) and l-glutamine (2 mM). Cells were routinely checked for mycoplasma (MycoAlert Lonza LT07-218). HLA typing was performed by Addenbrooke’s Hospital Histocompatibility and Immunogenetics (Tissue Typing) laboratory. Human embryonic kidney 293T and bladder carcinoma EJ28 cells were also grown in supplemented RPMI-1640 medium. Human foreskin fibroblasts HFF-T and fetal lung MRC-5T, both immortalized by expression of hTERT, the catalytic subunit of human telomerase (a gift of Stephen Graham, Department of Pathology) were grown in supplemented DMEM medium.

Cells (1 × 10^5^) growing in six-well plates were transfected with DNA expression constructs using Fugene (Promega). For RT-PCR, total RNA was prepared from cultured cells using RNAeasy mini (Qiagen) and Superscript III (Invitrogen) used to produce first strand cDNA. Amplification was carried out using 2× BiomixRed Taq polymerase (Bioline). RT-PCR products were analyzed by gel electrophoresis and cloned using Zero-Blunt Topo (Invitrogen).

Killing assay was by propidium iodide (PI) dye exclusion in cells treated with doxorubicin DOX (Cell Signaling). Cells were harvested and stained in 100 µl PBS (2.5 µg/ml PI) for 5 min before washing and analysis by fluorescence activated cell sorting (FACS) on a BD FACScalibur and data points collected on FL3/FSC. Inhibitors MG132 (5 µM for 5 h) and bafilomycin A (bafA) (5 µM 10 h) were used in cell assays. For activation, supernatants were collected from 24 h cocultures of Vγ9/Vδ2 T and HeLa-M cells containing 10 nM HMB-PP (Echelon) and used at a 1/10 dilution for 24 and 48 h.

### Immunoblot and Quantitative Mass Spectroscopy of Surface-Biotinylated Proteins

Cell lysates were prepared in buffer (50 mM Tris–Cl pH 7.5, 150 mM NaCl, 1% Triton-X, EDTA-free protease inhibitor, Roche) by incubation for 10 min at 4°C, then pre-cleared by centrifugation. For immune blots, cleared lysates were solubilized in SDS-PAGE buffer (5 min, 95°C) and separated in 10% SDS-PAGE gels (NextGel) then transferred to PVDF membrane. Antibodies were added directly to blocked membranes (5% Marvel/PBS 0.1% Tween 20) and incubated for 1 h with primary and HRP-conjugated secondary antibodies. Blots were visualized with ECL reagent (GEhealthcare) before exposure to X-ray film (Fuji). Monoclonal CD277 20.1 (LifeSpanBio), NRF2 (AF3925 R&D Systems), ABCG2 (ab108312) anti-calnexin (3811-100), and anti-GFP (SAB4301138) were used as well as 056 and B6 rabbit anti-B30.2 polyclonal sera at concentration between 0.5 and 1 µg/ml (30). Cell fractionation was carried out using Qproteome cell compartment kit (Qiagen).

For plasma membrane profiling (PMP), HeLa-M cells (5 × 10^7^) in large T175 flasks were either left untreated or treated with HMB-PP (10 nM for 10 h). Cells were washed (1× PBS) then biotinylation reagents added (30 min at 4°C). After quenching, cells were harvested by scraping and processed for immunoprecipitation, by lysis in 1% Tx-100 TBS pH 8 buffer with protease inhibitor (Complete EDTA free, Roche) and end-over-end rotation for 30 min at 4°C. After centrifugation (10,000 *g* for 10 min), Streptavidin agarose resin (Thermo Fisher) was added to supernatants, with incubation for 2 h at 4°C. Agarose beads were washed (20×) in lysis buffer, 20× in 0.5% SDS in PBS, 20× in 6 M urea/triethylammonium bicarbonate (TEAB) pH 8.5, 5× in TEAB pH 8.5 before digestion overnight with 0.5 µg trypsin in a final volume of 50 µl TEAB (31). Resulting peptides were analyzed by LC-MS/MS analysis using an Orbitrap Fusion instrument (Thermo Fisher) utilizing a 60-min gradient. Raw data were searched using MASCOT from within Proteome Discoverer (Thermo Fisher) v2.0 against the Uniprot human reference proteome. Peptide identifications were controlled at 1% FDR using Mascot Percolator. Proteins were quantified in a label-free manner using the precursor ion quantifier node.

### T Cell Assays

Ethical approval for working with blood samples from healthy donors was obtained from the South East Wales Local Ethics Committee (08/WSE04/17) and the Cambridge Local Ethics Committee (HBREC.2015.27). All volunteers provided written informed consent.

Vγ9/Vδ2 T cells were expanded from peripheral blood mononuclear cells of healthy donors with 1 µM zoledronate (Zometa; Novartis) and 50 U/ml IL-2 (Proleukin, Chiron) for 14 days and further enriched to purities >98% CD3+ Vγ9+ by negative selection using a modified human γδ T cell isolation kit that depletes B cells, αβ T cells, NK cells, dendritic cells, stem cells, granulocytes, and monocytes (Stem Cell Technologies). Unless otherwise stated, target HeLa cells were pretreated with 10 µM zoledronate or 10 nM HMB-PP, then washed extensively before coculture with γδ T cells at a ratio of 1:10 (10^4^ target: 10^5^T effector cells). The amount of IFN-γ secreted into the culture supernatant over 24 h was measured by ELISA (eBioscience). Mobilization of CD107a onto the cell surface over the first 5 h of coculture was determined using a PE-conjugated anti-CD107a antibody (H4A3; BD Biosciences) in the presence of monensin at a 1:2,000 dilution (GolgiStop). Cells were acquired on a FACS Canto II and analyzed with FlowJo. HeLa cells were also incubated with agonist CD277 20.1 monoclonal antibody (eBioscence) to induce activation independently of phosphoantigen.

The murine T cell hybridoma 53/4r/mCD28 TCR MOP was used to test Vγ9/Vδ2 TCR mediated activation, as described (32). In these experiments, HeLa cells used as stimulators (10^4^) were seeded in 96-well flat bottom tissue culture plate, allowed to adhere for 1 day before addition of T cells in fresh culture medium. Production of mouse IL-2 in culture supernatants was tested with a commercial mouse IL-2 ELISA kit.

## Results

### Two Haplo-Identical HeLa Cell Lines, HeLa-L and HeLa-M, Show Marked Variation in Their Ability to Activate Vγ9/Vδ2 T Cells *via* BTN3A

**Figure 1:**
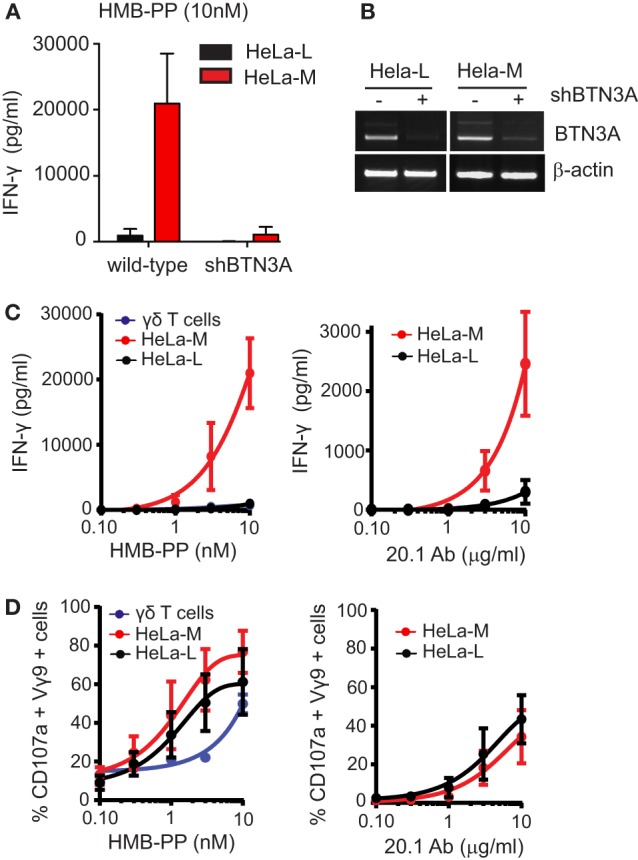
HLA haplo-identical HeLa-L and HeLa-M cells vary in their ability to present phosphoantigens to Vγ9Vδ2 T cells. **(A)** Vγ9/Vδ2 T cell activation as detected by production of IFN-γ. Wild-type and shRNA^BTN3A^ HeLa-L and HeLa-M cells were pretreated with hydroxy-3-methyl-but-2-enyl pyrophosphate (HMB-PP) (10 nM for 5 h) before addition of expanded Vγ9/Vδ2 T cells at E:T ratio of 10:1. After overnight incubation, culture supernatants were analyzed by ELISA. Bar chart shows mean and SD from triplicate measurements using T cells from one donor and is representative of three independent experiments using T cells from separate donors. **(B)** Knockdown of BTN3A expression by shRNA expression in HeLa cells. Transcripts for BTN3A and β-actin were amplified by RT-PCR from wild-type and shRNA^BTN3A^ HeLa-L and HeLa-M cDNA template and products analyzed by gel electrophoresis. PCR results shown are representative of three independent experiments. **(C)** Activation of Vγ9/Vδ2 T cells detected by ELISA for IFN-γ as in **(A)** using a concentration gradient of HMB-PP, agonist antibody CD277 20.1 and T cells expanded from two separate donors. Activation was induced by pretreatment of HeLa cells using HMB-PP for 5 h (left panel) and anti-BTN3A monoclonal antibody 20.1 for 30 min (right panel). Graphs show mean and SD from *n* = 2 and are representative of two independent measurements. **(D)** Activation of Vγ9/Vδ2 T cells as detected by flow cytometry for percent double positive Vγ9 and CD107a cells after coculture experiments as in **(C)**.

Various tumor-derived and primary epithelial cell lines were tested for their ability to respond to the aminobisphosphonate drug zoledronate and present the microbial compound HMB-PP to Vγ9/Vδ2 T cells (Figure S1A in Supplementary Material). Two HeLa cervical epithelium carcinoma cell lines, HeLa-L and HeLa-M, showed profound differences in their ability to elicit cytokine (Figure 1; Figure S1 in Supplementary Material). HeLa-M cells were far more potent than HeLa-L cells in stimulating release of IFN-γ (Figure 1A; Figure S1B in Supplementary Material) and TNF-α (Figure S1C in Supplementary Material) upon pretreatment with HMB-PP. Responses to zoledronate similarly differed between the two cell lines (data not shown). Responses to either HeLa cell line were abrogated by expression of a single shRNA targeting the three BTN3A isoforms (shRNA^BTN3A^ cells), confirming the importance of BTN3A in mediating Vγ9/Vδ2 T cell responses to both HMB-PP and zoledronate (Figures 1A,B).

The differential responsiveness of Vγ9/Vδ2 T cells to HeLa-L and HeLa-M cells was replicated when using the pan-BTN3A specific agonist antibody CD277 20.1, which induces BTN3A-dependent activation of Vγ9/Vδ2 T cell activation in the absence of HMB-PP or zoledronate (Figure 1C). In addition, production of IL-2 from a murine T cell hybridoma expressing human Vγ9/Vδ2 transgenes showed a similar differential response to HMB-PP (Figure S1D in Supplementary Material).

In striking contrast to these findings of cytokine secretion as a functional read-out for T cell responses, surface mobilization of CD107a/LAMP1 did not differ between Vγ9/Vδ2 T cells responding to the two HeLa lines, upon HMB-PP or CD277 20.1 antibody pretreatment (Figure 1D), indicating differential signaling requirements for the induction of cytokine secretion versus CD107a mobilization.

Taken together, these experiments demonstrated that HeLa-M cells were more efficient at activating Vγ9/Vδ2 T cells *via* BTN3A than HeLa-L cells, with regard to the induction of cytokine expression but not CD107a mobilization. These cell lines, therefore, represent a useful experimental model to investigate the molecular mechanism underlying the specific Vγ9/Vδ2 T cell responses to endogenous and exogenous pAg.

### BTN3A Transcripts Are Identical in HeLa-L and HeLa-M

**Figure 2:**
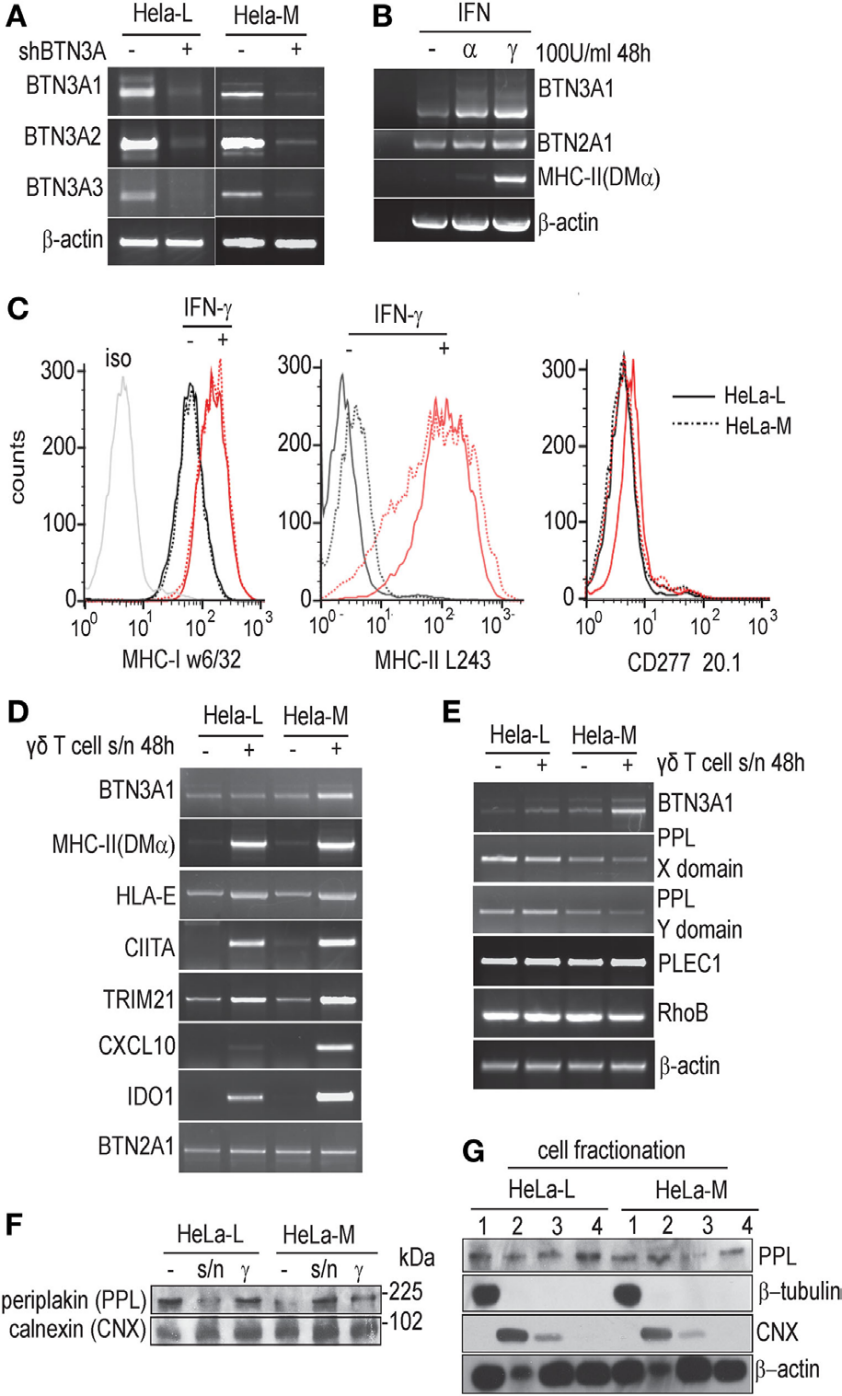
Transcripts for the three BTN3A isoforms are unchanged in HeLa-L and HeLa-M. **(A)** Amplification of full-length BTN3A1, BTN3A2, and BTN3A3 transcripts by RT-PCR from wild-type and shRNA^BTN3A^ HeLa-L and HeLa-M cDNA template. **(B)** RT-PCR for BTN3A1, BTN2A1, and MHC-II (DMα) from cDNA derived from HeLa-M cells either left untreated or treated with IFN-α or IFN-γ. RT-PCR products were analyzed by gel electrophoresis, with β-actin as loading control. Results are representative of three experiments. **(C)** Analysis of activation markers MHC-I (w6/32), MHC-II (L243), and BTN3A (CD277 20.1) by flow cytometry in HeLa cells treated with or without IFN-γ. Results representative of triplicate experiments. Red lines indicate IFN-γ treated and black lines untreated samples. Solid lines are HeLa-M and dotted lines HeLa-L. **(D)** Expression of activation markers as detected by RT-PCR in HeLa-L and HeLa-M cells left untreated or treated with γδ T/HeLa-M cell coculture supernatant collected as described (see Materials and Methods). Transcripts for BTN3A1, HLA-E, DMα, CIITA, TRIM21, CXCL10, IDO-1, and BTN2A1 were amplified and visualized by gel electrophoresis. Results are representative of duplicate experiments. **(E)** Expression of the BTN3A1 interacting molecules protein periplakin (PPL) and RhoB in treated and untreated HeLa-L and HeLa-M cells analyzed by RT-PCR, as in **(C)**, with transcripts for β-actin as a loading control. Results are representative of duplicate experiments. **(F)** Lysates from HeLa-L and HeLa-M cells left untreated or treated with γδ T/HeLa-M cell coculture supernatant or IFN-γ, were analyzed by western blot for expression of PPL protein. Duplicate blots were probed with calnexin (CNX) antibody as a loading control. **(G)** Cell fractionation to compare the sub-cellular distribution of PPL protein in HeLa-L and HeLa-M cells. Fractions for soluble (1), membrane (2), nuclear (3), and cytoskeletal/detergent resistant membrane (4) fractions were prepared and analyzed by western blot using anti-PPL, β-tubulin, CNX, and β-actin antibodies.

In order to account for the functional differences between HeLa-L and HeLa-M cells, full-length BTN3A transcripts were amplified by RT-PCR from HeLa cDNA, cloned, and sequenced (Figure 2). As shown in Figure 2A, transcripts of all three BTN3A isoforms were expressed at comparable levels in both HeLa-L and HeLa-M, with no differences in splicing and all were targeted with equal efficiently by shRNA. No sequence variation or mutation was detected when six clones from each cell line for each BTN3A isoform were compared to consensus sequences (BTN3A1 NM_007048, BTN3A2 NM_007047, BTN3A3 NM_006994) (data not shown). Known synonymous SNPs common in sub-Saharan Africa, but rare in other populations, were identified in all BTN3A1 sequences (dbSNP rs3857550, rs2393650, and rs537072716), suggesting that both HeLa lines were homozygous for these polymorphisms. BTN3A2 and BTN3A3 nucleotide sequences showed no polymorphisms; however, allelic exclusion or haplotype bias in transcript expression was not assessed. By tissue typing of genomic DNA, both HeLa cell lines were HLA-A68-B15:03/15 -Bw6 -C12 -DR 01:02/01:2601 haplo-identical. These data provided convincing authentication of the HeLa lines by conformation of their common genetic origin.

### Cellular Markers of Activation Are Unaffected

Transcripts for BTN3A1 were shown to be induced by type I and type II interferons and HeLa-L and HeLa-M cells did not differ in this regard (Figure 2B and data not shown). By cytometry, there was comparable upregulation of surface MHC-I and MHC-II by IFN-γ treatment; however surface staining using CD277 20.1 antibody was not detected, indicating a discrepancy between transcriptional regulation and protein expression (Figure 2C).

In order to investigate the possibility of a soluble factor influencing T cell activation and also a more physiologically relevant stimulus potentially influencing BTN3A1 expression, we studied the response of both HeLa lines to treatment with supernatants collected from 24 h cocultures of Vγ9/Vδ2 T cells activated with HMB-PP (10 nM) loaded HeLa-M cells. Transcripts for a number of activation markers, including MHC-II DMα, HLA-E, MHC-II trans-activator CIITA, and TRIM21 were analyzed by RT-PCR in addition to BTN3A1 (Figure 2D). Some variation in HeLa-derived transcripts was detected, particularly CXCL10 and IDO-1 and a slight increase in BTN3A1 transcripts upon activation in HeLa-M was not seen in HeLa-L. However, convincing BTN3A expression or activation at the cell surface was not detected by cytometry using CD277 20.1 antibody as for Figure 2C (data not shown). BTN2A1 transcripts did not vary between samples, and there was no correlation between butyrophilin BTN2A or BTN3A expression and CIITA/MHC-II in HeLa cells (6). A more detailed quantitative analysis of some transcripts, for example CXCL10 or IDO-1 may be warranted, but the data so far were not consistent with a major signaling defect in either HeLa line which could account for differential responses in T cell assays or with transcriptional control affecting BTN3A1 activation.

### No Variation in Expression of BTN3A1 Interactors

The cytoskeletal adaptor protein periplakin (PPL) and the RAS-superfamily GTPase RhoB were recently identified as BTN3A1 interacting molecules (30, 33). We tested whether the differential BTN3A1-dependent responsiveness of Vγ9/Vδ2 T cells to HMB-PP loaded HeLa-L and HeLa-M might be due to variation in PPL and/or RhoB expression. In duplicated RT-PCR experiments, transcripts for PPL were slightly more abundant in HeLa-L compared to HeLa-M. By analyzing separate domains of the mRNA, no variation in splicing which conceivably could affect protein interactions or shRNA-mediated transcript suppression was detected (Figure 2E). In addition to PPL itself, we detected transcripts for the PPL interacting molecule PLEC1 (Figure 2E) but not for kazrin or envoplakin (34–36). RhoB transcripts were detected abundantly in both HeLa lines and were not obviously affected by activation with γδ T cell/HeLa-M coculture supernatants. By western blot, major variation in PPL protein level was not detected between the HeLa cell lines either with, or without, activation using IFN-γ or using T cell supernatants (Figure 2F). Neither did we see any discrepancy in cellular distribution by cell fractionation; PPL was distributed equally in soluble, membrane, nuclear, and cytoskeletal/detergent resistant membrane fractions as shown previously (Figure 2G) (30).

These experiments suggested that differences in the expression of BTN3A1 interacting molecules did not account for the functional variation between HeLa-L and HeLa-M cells. By contrast, in a direct comparison in T cell assays of HeLa-L and HeLa-M PPL knockdown lines, produced by expression of three different PPL targeting shRNA (Figure S2 in Supplementary Material), wide variation in responses as measured by IFN-γ was detected. The PPL knockdown experiments were consistent with our initial conclusion of a regulatory rather than mandatory role for PPL in transmitting activation signals to T cells (30).

### Detection of BTN3A1 by Re-Expression in shRNA^BTN3A^ Cells

**Figure 3:**
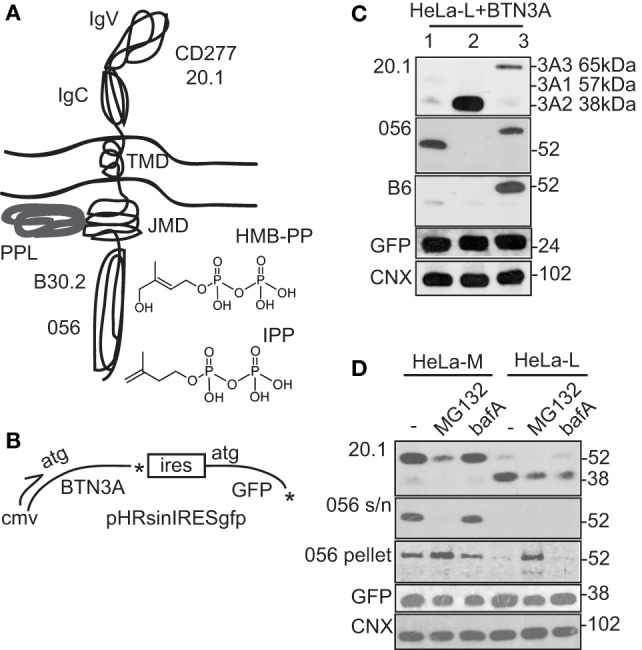
Detection of BTN3A1 by re-expression in shRNA^BTN3A^ cells. **(A)** Diagram of BTN3A1 protein and structure of the two principal stimulatory phosphoantigens (pAg) hydroxy-3-methyl-but-2-enyl pyrophosphate (HMB-PP) and isopentenyl pyrophosphate (IPP). Sites of pAg interaction in the BTN3A1 B30.2 domain, protein periplakin (PPL) interaction at the juxta-membrane domain (JMD) and the antibodies used for detection are highlighted. BTN3A3 has a structure similar to BTN3A1, whereas BTN3A2 is truncated after the JMD and lacks the B30.2 domain. **(B)** Diagram of BTN3A pHRsinIRESgfp lentiviral expression vector used in re-expression studies. Independent measure of transgene expression is provided by internal ribosome entry site (IRES) driven green fluorescent protein (GFP). **(C)** Western blot of HeLa-L cells transiently transfected with expression vectors for BTN3A1, BTN3A2, and BTN3A3. Monoclonal anti-BTN3A (CD277 20.1) antibody and polyclonal sera 056 and B6, directed to B30.2 domains of BTN3A1 and BTN3A3, respectively, were used. Anti-GFP and calnexin (CNX) antibodies provided loading controls for expression of transgene and endogenous proteins, respectively. **(D)** Western blot analysis of stable BTN3A1 re-expression lines of HeLa-L and HeLa-M shRNA^BTN3A^ cells, either left untreated or treated with MG132 or bafilomycin A (bafA). Anti-CD277 20.1 antibody and polyclonal sera 056 were used, together with anti-GFP and CNX control antibodies as in **(C)**. Detection of CD277 20.1 and 056 reactive protein bands required extended exposure to X-ray film.

Although mRNA transcripts were detectable, BTN3A proteins were not abundantly expressed in HeLa cells. In addition, CD277 20.1 antibody binding to HeLa cells was not convincing by cytometry (Figure 2C), despite the fact that this agonistic antibody elicited potent Vγ9/Vδ2 T cell activation (Figure 1B). In order to overcome the limitations posed by low expression levels, we re-expressed BTN3A isoforms in wild-type and BTN3A knockdown (shRNA^BTN3A^) cells using a lentiviral vector which afforded an independent marker of transgene expression via detection of GFP (Figures 3A,B).

By transient transfection of either HeLa-L or HeLa-M with expression vectors for BTN3A1, BTN3A2, and BTN3A3, significant surface CD277 20.1 antibody staining was not detected for any of the three BTN3A isoforms, even with high GFP transgene expression (Figure S2A in Supplementary Material). In contrast, BTN3A2 and BTN3A3, but not BTN3A1, were productively expressed on the surface of 293T cells using the same transfection strategy, indicating that surface expression of BTN3A/CD277 was tightly regulated, by a mechanism which targeted BTN3A1 preferentially and which differed between cell lines.

By western blot analysis of lysates from transiently transfected cells, there was marked variation in the ability of CD277 20.1 antibody to detect the different BTN3A isoforms (Figure 3C; Figure S2B in Supplementary Material). While BTN3A1 was barely detectable, BTN3A2 was abundant (at levels expected from transgene expression) and BTN3A3 was expressed at intermediate levels. Authentic expression of full-length proteins was confirmed in some experiments using isoform specific antisera directed against the B30.2 domains of BTN3A1 and BTN3A3 (Figure 3C; Figure S2 in Supplementary Material), requiring extended exposure to X-ray film.

The above experiments indicated an intracellular checkpoint to BTN3A1 expression. To determine if this was mediated by protein degradation, we tested the effect of the proteasome inhibitor MG132 and bafA, an inhibitor of lysosome/autophagy pathways, on BTN3A1 expression in lentivirally transduced HeLa-L and HeLa-M shRNA^BTN3A^ cells (Figure 3D). By western blot using CD277 20.1 antibody, full-length soluble BTN3A1 (57 kDa) was detected in the untreated HeLa-M re-expression line, but was barely detectable in the HeLa-L lysate. Instead, a lower molecular weight band of 38 kDa was detected in HeLa-L samples, indicative of BTN3A1 protein cleavage. Alternatively, the 38-kDa protein could represent endogenous BTN3A2, although BTN3A2 was not detected abundantly in un-transfected cell lines (Figure S3 in Supplementary Material). Levels of detergent-soluble, full-length BTN3A1 were reduced by MG132 treatment, but were unaffected by bafA. In contrast, MG132 treatment of HeLa-L cells led to the appearance of full-length BTN3A1 (57 kDa) in the detergent resistant fractions from Tx-100 lysates, suggesting a potential redistribution of BTN3A1 in the membrane by proteasome inhibition.

Taken together, these results were consistent with a mechanism controlling surface expression of BTN3A protein isoforms, in particular BTN3A1, by protein production, stability, or trafficking.

### PMP of HMB-PP Treated Cells Identifies ABCG2

We next searched for differences in cellular factors that might influence pAg presentation in the two HeLa cell lines using surface biotinylation, followed by immunoprecipitation and quantitative mass spectroscopy (37). Bulk HeLa-M cells were allowed to adhere (10 h), then were either left untreated or treated with HMB-PP (10 nM for 10 h) prior to PMP as described (38).

**Figure 4:**
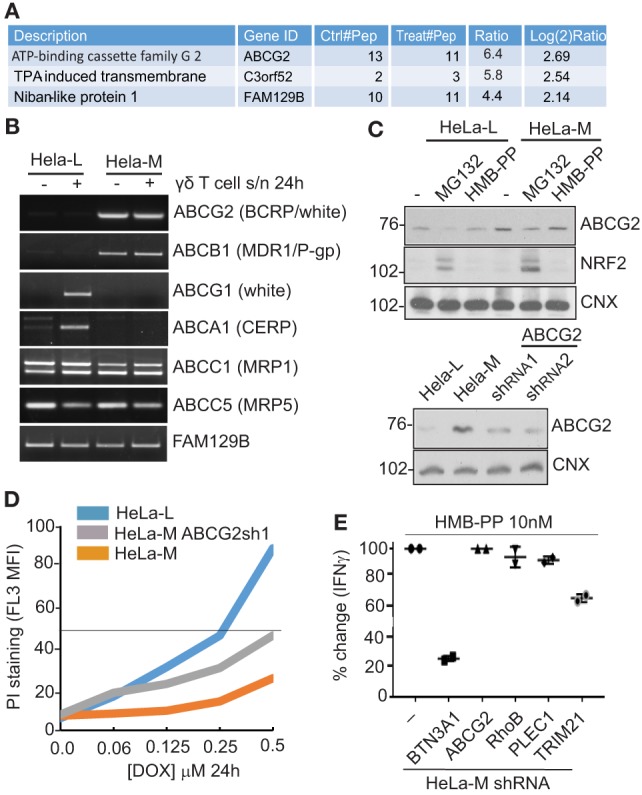
Plasma membrane profiling (PMP) by mass spectroscopy identifies ABCG2. **(A)** Table of top hits identified using PMP to compare HMB-PP treated and untreated HeLa-M cells. Several independent peptides of ATP-binding cassette (ABC) transporter ABCG2 were detected, with modest increase (log_2_ 2.69) by treatment. Other potential candidates by these criteria (C3orf52 and FAM129B) are listed and were less significant. **(B)** Dysregulation of a subset of ABC transporters, including ABCG2, in HeLa cells detected by RT-PCR. Template cDNA was prepared from total RNA from HeLa-L and HeLa-M cells left untreated or treated with γδ T cell supernatant for 24 h. RT-PCR products were analyzed by gel electrophoresis. **(C)** Variation in ABCG2 protein expression in HeLa cells by western blot. Top panel: HeLa-L and HeLa-M cells were left untreated or treated with MG132 (5 µM for 10 h) or HMB-PP (10 nM for 10 h). Cell lysates were prepared and analyzed using antibodies directed to ABCG2, NRF2, and calnexin (CNX), which acts as a loading control. Bottom panel: western blot analysis of shRNA^ABCG2^ cells confirms reduction in ABCG2 protein expression. **(D)** HeLa-M cells show ABCG2-dependent resistance to cytotoxic drug doxorubicin (DOX). Wild-type HeLa-M, HeLa-L, and shRNA^ABCG2^ HeLa-M cells were incubated (24 h) with DOX at the indicated concentrations. Cells were stained using propidium iodide (PI) (2.5 µg/ml for 5 min) as a marker of plasma membrane integrity. After washing, cells were analyzed by cytometry. Chart shows mean fluorescence intensity (MFI) of PI staining. **(E)** No effect on T cell activation using shRNA^ABCG2^ HeLa-M cells. The effect on the efficiency of Vγ9/Vδ2 T cell activation for a series of HeLa-M gene knockdown lines, including shRNA BTN3A1, ABCG2, RhoB, PLEC1, and TRIM21. Cells were pretreated with HMB-PP (10 nM for 6 h) before Vγ9/Vδ2 T cells from a single donor were applied. Coculture was allowed to proceed overnight when culture supernatants were analyzed by ELISA. Chart shows percent (%) change in detected IFN-γ relative to wild-type HeLa-M cells of duplicate determinations for each cell line.

By this approach, ABCG2 (also called breast cancer resistance protein) was identified as a candidate whose expression was significantly influenced by HMB-PP in HeLa-M cells (Figure 4A). ABCG2 is a member of the superfamily of ABC transporters and was originally characterized as a drug efflux pump (39). Variation in expression was modest (log2 = 2.69 increase), but several independent peptides of ABCG2 were detected. FAM129B, a molecule involved in RAS activation, was another candidate potentially affected by HMB-PP treatment (log2 = 2.15 increase) (40). By RT-PCR from the panel of untreated versus treated HeLa cDNA (Figure 4B), transcripts for ABCG2 were reduced in HeLa-L compared to HeLa-M samples, an observation not seen for other candidates, while FAM129B transcripts did not vary (Figures 2C,D). Reduced expression of ABCG2 total protein in HeLa-L compared to HeLa-M cell lysate was confirmed by western blot (Figure 4C), but variation in expression by HMB-PP treatment was not and surface ABCG2 was not detected by flow cytometry. However, reduced levels of ABCG2 by MG132 treatment was seen (Figure 4C), similar to that detected for BTN3A1 (Figure 3D). Expression of NRF2 (nuclear factor erythroid 2-related factor 2), a basic leucine zipper (bZIP) protein and major transcriptional regulator of ABCG2, was stabilized in the MG132 treated samples as expected, with increased levels of NRF2 evident in HeLa-M versus HeLa-L samples, consistent with the higher expression of ABCG2 in these cells (Figure 4C).

From these data, ABCG2 was the most promising candidate to account for the differential effects of the HeLa cell lines in T cell assays, implicating this molecule in pAg presentation. Therefore, HeLa-M cells expressing shRNA targeting ABCG2 (shRNA^ABCG2^) were constructed. Reduced ABCG2 expression in shRNA^ABCG2^ cells was confirmed by western blot (Figure 4C bottom panel) as was a functional effect in killing assays, by increased sensitivity to DOX, a major target substrate of ABCG2 (Figure 4D). However, in coculture assays with Vγ9/Vδ2 T cells, no effect on the efficiency of T cell activation of HMB-PP loaded HeLa-M shRNA^ABCG2^ cells was detected, as measured by ELISA for IFN-γ (Figure 4E). With shRNA^BTN3A^ cells acting as control, a modest effect of shRNA^TRIM21^ cells was detected in these experiments, but no effect of shRNA^RhoB^ or shRNA^PLEC1^ cells. RhoB has been implicated previously in pAg presentation using similar shRNA-mediated knockdown (33), so the lack of specific effects in our experiments may be indicative of ineffective transcript suppression or variation between different cell lines. The mechanism by which TRIM21 may be affecting γδ T cell activation is unexplained (41).

Expression of a number of functionally related ABC transporters was examined by RT-PCR in the activated HeLa cell panel, including ABCB1 (multidrug resistance 1, MDR1), ABCG1 (white), ABCA1 (cholesterol export regulatory protein, CERP), ABCC1 (multi-resistance protein 1), and ABCC5 (multi-resistance protein 5). While expression of the latter two (ABCC1 and ABCC5) did not vary, transcripts for ABCB1, ABCG1, and ABCA1 did (Figure 4B). ABCB1 showed a pattern of expression similar to ABCG2, while ABCG1 and ABCA1 transcripts were only detected in treated samples of HeLa-L, and not in HeLa-M.

Taken together, the results revealed dysregulated expression of a subset of ABC transporters, which correlated with efficiency of γδ T cell activation, suggesting a link between these members of the ABC protein superfamily and pAg-dependent γδ T cell activation.

## Discussion

Butyrophilin BTN3A proteins are the critical determinants of pAg presentation to Vγ9/Vδ2 T cells, but their precise role and how they are regulated is not fully understood. In order to address this issue and with a view to identifying novel effectors, we studied two HeLa cell lines which showed marked differences in their ability to activate Vγ9/Vδ2 T cells in response to pAg loading. Endogenous levels of all BTN3A isoforms were low in HeLa cells and were not detected by western blotting or flow cytometry. By PMP using quantitative mass spectroscopy in HeLa-M cells that were untreated or treated with HMB-PP, BTN3A1 and BTN3A2 isoforms were detected at low levels, with minor variation by treatment. Subsequent experiments using PMP may allow for interrogation of endogenous BTN3A proteins, their regulation by pAg and correlation with T cell activation in different presenting cells (42).

BTN3A1 protein stability at steady state, where BTN3A1 transgene re-expression was carefully controlled by GFP, was reduced significantly compared to that of the other isoforms, BTN3A2 and BTN3A3. We also detected differences in BTN3A1 protein between the two HeLa cell lines. The data are consistent with regulation of BTN3A1 at the level of protein stability. Protein truncation, presumably resulting from proteolytic cleavage to remove the B30.2 domain was also evident using anti-BTN3A antibodies. The possibility that posttranslational modification, for instance by N-linked glycosylation, may be influencing CD277 20.1 antibody recognition of BTN3A1 by affecting protein trafficking and stability cannot be ruled out. It is also unclear whether the free B30.2 domain, a potential degradation intermediate, has a separate function.

It is possible that specific miRNA or mRNA processing, resulting in a block or premature stop in translation could account for truncated and/or reduced protein levels, although no effect on BTN3A1 transcripts in re-expression lines was detected by RT-PCR. Since inhibitor MG132 did not give a clear picture of protein stabilization, as exemplified here by stabilization of NRF2, it is not clear whether BTN3A1 instability is due to proteasome function. Lysosomal inhibition also had no effect. Therefore, another mechanism of protein maturation and trafficking, acting at the level of protein translation or membrane insertion may be involved.

Control of BTN3A1 stability would provide a dynamic mechanism to control Vγ9/Vδ2 T cell activation, similar to that shown for the homologous molecule PD-L1 in controlling αβ T cell responses (43). The fact that BN3A1 expression appears to be limiting over BTN3A2 will also influence interactions between the two isoforms, proposed recently to be essential for Vγ9/Vδ2 T cell activation (11). Regulation of BTN3A1 stability would also offer a means to modulate immune-surveillance by Vγ9/Vδ2 T cells, representing a potential immune-evasion mechanism exploited by tumors and intracellular pathogens.

ABCG2 was identified as a potential candidate protein in pAg-dependent T cell activation using PMP of HMB-PP treated HeLa-M cells. ABCG2 correlates functionally with dye negative side population, a biomarker of cancer stem cells (44) and was considered an interesting functional candidate in pAg presentation because of the known association with statins, cholesterol metabolism, and the HMG-CoA reductase pathway (45, 46). However shRNA^ABCG2^ knockdown cells showed no functional defect in T cell assays. It is possible that shRNA did not reduce ABCG2 levels sufficiently in HeLa-M^ABCG2^ cells to influence this assay, or that other members of the ABC transporter family with similar expression and substrate profile (for example, ABCB1) compensated for its absence in these cells.

In addition to ABCG2 and ABCB1, transcription of ABCA1 and ABCG1 was dysregulated. ABCA1 and ABCG1 are also linked functionally to cholesterol metabolism, acting as regulators of cholesterol transport (47, 48). Since these molecules are not known to be induced by pro-inflammatory cytokines, it is unclear what signals from the γδ T cell supernatant, derived from cocultures with HeLa-M cells containing 10 nM HMB-PP, induced transcription of ABCA1 and ABCG1 in HeLa-L cells but not in HeLa-M. Published data describe a role in γδ T cell activation for ABCA1 acting together with BTN3A1 as a pAg efflux transporter in myeloid cells, linked to the LXR (liver X receptor) transcription factor (49). In the ABCA1 efflux model (49), ABCA1 drives IPP into the extracellular compartment to activate γδ T cells *in trans*. In our system, ABCA1 expression in activated HeLa-L cells would theoretically reduce intracellular HMB-PP concentration to thereby limit the pool available to activate BTN3A1. However, ABCA1 did not enhance T cell activation by HMB-PP-loaded HeLa-M because it did not appear to be expressed in these cells. Therefore, ABCA1 alone cannot account for the effects we detected, and it is also difficult to resolve an extracellular pAg delivery and sensing mechanism with the binding of pAg to the cytosolic B30.2 domain of BTN3A1 (9), unless this interaction promotes pAg export to bind BTN3A1 externally, as proposed by other workers (7, 50).

Conventional MHC restricted antigen presentation to TCR expressed on αβ T cells relies on peptide transport by the heterodimeric TAP1/2 transporter, and it is plausible that pAg transport similarly requires ABC family members. Other workers have investigated ABCC5 (51) in addition to ABCA1 (49). Although most are described as drug or xenobiotic efflux pumps contributing to a multidrug resistance phenotype, endogenous substrates for many ABC transporters are largely uncharacterized. If these ABC transporters are involved, evidently there is more complexity in this system than current models of pAg presentation allow, in particular whether pAg transport is into or out of cells (or both) (50, 52) and whether they influence BTN3A expression by direct interaction (49). Further analysis of these ABC transporters seems warranted.

HeLa cells were the first cell line to be used for biomedical research, originating in the 1950s (53). HeLa-L and HeLa-M are two sublines the origins of which are obscure but which our data so far indicate are genetically identical. The cell lines were free of mycoplasma or other detectable infection and the chance observation of differences in pAg presentation and interaction with expanded Vγ9/Vδ2 T cells derived from different donors were stable for over 3 years with repeat samples acquired from original sources. The HeLa-M line historically showed elevated responses to IFN-γ (54) and in our hands showed resistance to the anticancer drug DOX in dye-exclusion killing assay, an effect reversed to some extent by ABCG2 knockdown. A feasible scenario, therefore, would be selection by an anticancer drug such as DOX resulting in DNA mutation (55), permanent activation of the KEAP1/NRF2 stress response pathway and increased ABCG2 expression in these cells (56). A similar approach was used to identify ABCG2 from a drug-resistant MCF-7 line (57).

Alternatively, signaling defects of the hedgehog pathway or LXR transcriptional regulation may be involved in ABC transporter expression (47, 58–60). If DOX impacts regulation of BTN3A-dependent pAg sensing, then the use of such cytotoxic drugs in cancer treatment could impact immunotherapy strategies based on γδ T cells (23) by selecting clones with increased expression of survival factors which promote tumor growth. In any event, expression of this subset of ABC transporters may act as a biomarker for enhanced Vγ9/Vδ2 T cell activation by cytokine secretion, and it will be interesting to test this correlation in other cells and with other markers of activation. Current experiments are underway to identify a more comprehensive catalog of differences between HeLa-L and HeLa-M cells.

## Author Contributions

DR, ME, and TH designed research; DR, H-CC, JW, AH, JY, SS, HR, and TH performed research; DR, ME, H-CC, and TH analyzed data, JT and PL provided capacity; DR and ME wrote the paper with input from all authors.

## Conflict of Interest Statement

The authors are not aware of any financial holdings or other personal and professional relationships which could be construed as affecting the objectivity of this manuscript. The handling Editor declared a past co-authorship with one of the authors (ME).
