# Supplementary material for: Regulation of Human γδ T Cells by BTN3A1 Protein Stability and ATP-Binding Cassette Transporters
Source: Front Immunol. 2018 Apr 4;9:662. doi: 10.3389/fimmu.2018.00662 (PMC5893821; doi:10.3389/fimmu.2018.00662)
Supplement: Supplementary file 1 [file presentation_1.PDF]

Supplementary Table I. Sequences of DNA oligonucleotide primers used in this study.

NAME	SEQUENCE	COMMENT
3A1.FOR<br>3A1.REV	ATGAAAATGGCAAGTTTCCTGG<br>AAGCTTCGCTGGACAAATACTCAGGGCC	RT-PCR and cloning<br>NM_007048
3A2.FOR<br>3A2.REV	GAATTCCATAGATGAAAATGGCAAGTT<br>GATATCGGCTGACTTATTGGTATCG	NM_007047
3A3.FOR<br>3A3.REV	GAATTCCATAGATGAAAATGGCAAGTT<br>AAGCTTGTAAAGTGCTTCAGTGCGTGCC	NM_006994
BTF3.4.F2<br>BTF3.R2<br>BTF4.R2	TAGATGAAAATGGCAAGTTCCC<br>GTAAAGTGCTTCAGTGCGTGCC<br>GGCTGACTTATTGGTATCGGACG	RT-PCR
BT3A.F<br>BT3A.R	ATCCCGGCTGTGGAAGCACCTGTGG<br>TCCAGTCTCATAGTCCAGGAAG	Universal BTN3A<br>RT-PCR primers
β-2M.F<br>β-2M.R	GGATCCCGCGATGTCTCGCTCCGTGG<br>GCGGCCGCTTACATGTCTCGATCCCAC	RT-PCR and cloning<br>NM_004048
ACTIN F<br>ACTIN R	GTGTAACGCAACTAAGTCATAGTC<br>CATGGATGATGATATCGCCG	NM_001101
TOP2A.F<br>TOP2A.R	CATGGAAGTGTCACCATTGCAGC<br>GCTGTCCAAATATGAGAGCTGG	NM_001067
RHOB.F<br>RHOB.R	CGCTCATGGCGGCCATCCGC<br>GCCCTCATAGCACCTTGCAGC	NM_004040
IDO1.F<br>IDO1.R	CACGCTATGGAAAACTCCTGG<br>CTTTGGGTCTTCCCAGAACCC	NM_002164
C2TA.F<br>C2TA.R	ATGCGTTGCCTGGCTCCACGCCC<br>CTTCCAGTGCTTCAGGTCTGCCGG	NM_000246
CXCL10.F2<br>CXCL10.R2	CTGCCATTCTGATTTGCTGCC<br>GGAGATCTTTTAGACCTTTCC	NM_001565
CXCL9.F<br>CXCL9.R	CTCTTGGGCATCATCTTGCTGG<br>GTAGTCTTCTTTTGACGAGAACG	NM_002416
CXCL11.F<br>CXCL11.R	GTGAAGGGCATGGCTATAGCC<br>CAACTTTTTGATTATAAGCC	NM_005409
GBP5 F<br>GBP5 R2	CCTACCTGATGAACAAGCTGGC<br>GGTCCAGCAGCTCCTGGAGGG	NM_052942
HLA-E.F<br>HLA-E.R	GGGATCATGGTAGATGGAACCC<br>CAAGCTGTGAGACTCAGACCCC	NM_005516
PPL.F2<br>PPL.R2	GATATCCTCAAGTACCGCCGGGAG<br>GGATCCGGCCTGGATGAAGGCTTC	RT-PCR XW domain<br>NM_002705 1261-1818
PPL.F6<br>PPL.R6	GCTGCAGAAGAATGCCGACCAGG<br>GCGCAGGTTGGTCACACGCTCC	RT-PCR exon 2<br>NM_002705 189-444
ABCG2.F<br>ABCG2.R	CAGATGTCTTCCAGTAATGTCG<br>CTCCTCCAGACACACCACGGA	RT-PCR<br>NM_004827
ABCA1.F<br>ABCA1.R	AGCTGAGGTTGCTGCTGTGG<br>CAGTTTCTCCCTTGGTAGGCC	RT-PCR<br>NM_005502
ABCC5.F<br>ABCC5.R	GTGTGAGGGAGAGAACCAGC<br>GACAGAGACCACACGTCTTCC	RT-PCR<br>NM_005688

PLEC1sh1.F<br><br>PLEC1sh1.R	GATCCGTCCACAAGCTGCAGAATGTTTCAAGAGAA CATTCTGCAGCTTGTGGATTTTTTG<br>AATTCAAAAAATCCACAAGCTGCAGAATGTTCTCT TGAAACATTCTGCAGCTTGTGGACG	shRNA NM_000445
ABCG2sh1.F<br><br>ABCG2sh1.R<br><br>ABCG2sh2.F<br><br>ABCG2sh2.R	GATCCGTGGAAACTGCTGCTTTAGTTCAAGAGACT AAAGCAGCAGTTTCCACTTTTTTG<br>AATTCAAAAAAGTGGAAACTGCTGCTTTAGTCTCT TGAACTAAAGCAGCAGTTTCCACG<br>GATCCGGATCATGAAACCTGGTCTTTCAAGAGAAG ACCAGGTTTCATGATCCTTTTTTG<br>AATTCAAAAAAGGATCATGAAACCTGGTCTTCTCT TGAAAGACCAGGTTTCATGATCCG	shRNA NM_004827
TR21sh.F<br><br>TR21sh.R	GATCCGCAGCACGCTTGACAATGATTCAAGAGATC ATTGTCAAGCGTGCTGCTTTTTTG<br>AATTCAAAAAAGCAGCACGCTTGACAATGATCTCT TGAATCATTGTCAAGCGTGCTGCG	Mallory et al 2010
RhoBsh1.F<br><br>RhoBsh1.R	GATCCGAAGCACTTCTGTCCCAATTTCAAGAGAAT TGGGACAGAAGTGCTTCTTTTTTG<br>AATTCAAAAAAGAAGCACTTCTGTCCCAATTCTCTT GAAATTGGGACAGAAGTGCTTCG	shRNA NM_004040

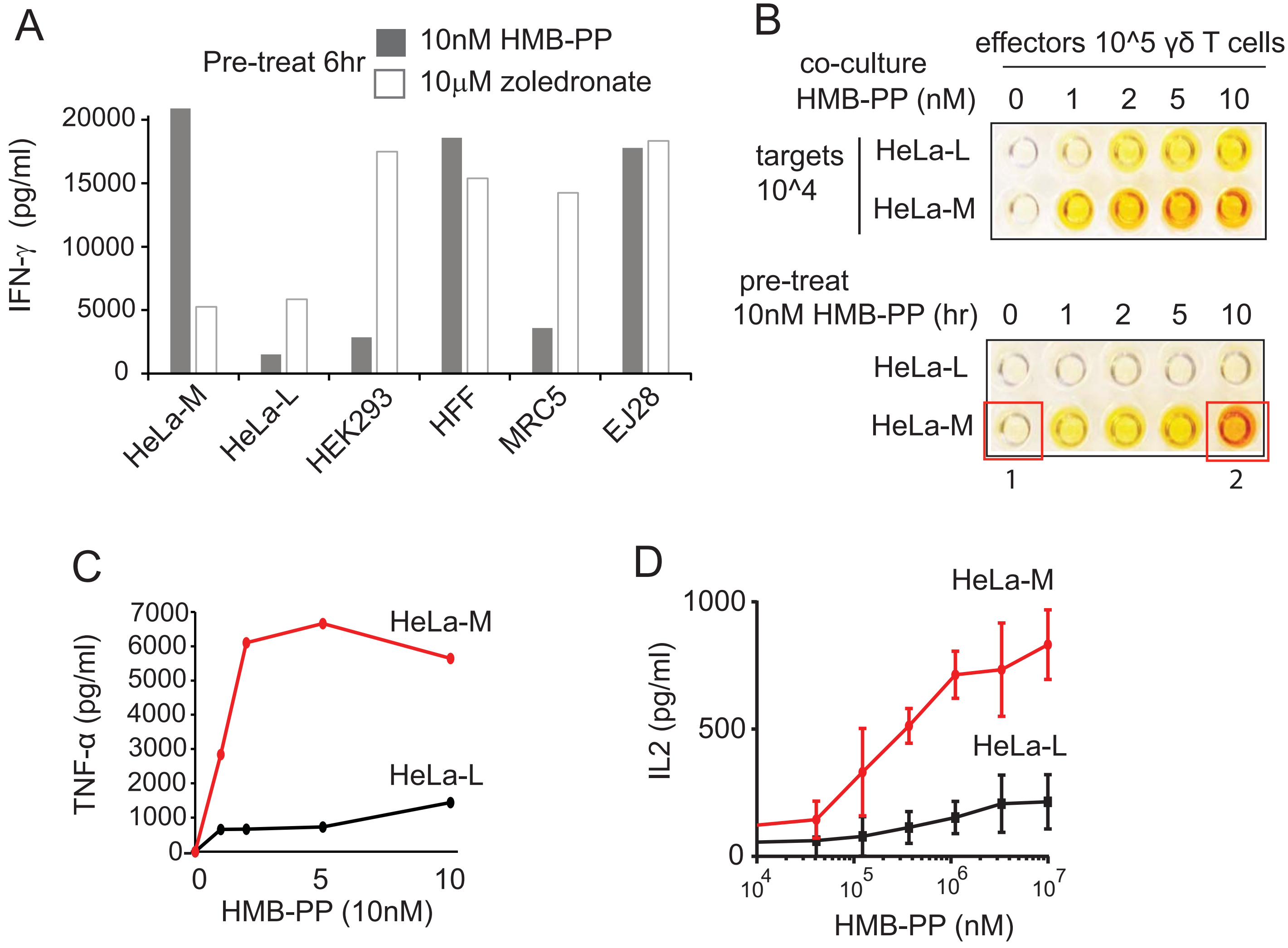

Supplementary Figure 1

(A) T cell activation as detected by production of IFN-γ. A series of human tumour derived and immortalized normal human epithelial cell lines were pre-treated with HMB-PP (10 nM for 5 h) or zoledronate (10 μM 5 h) before addition of purified Vγ9/Vδ2 T cells at E:T ratio of 10:1. After overnight incubation co-culture supernatants were analysed by ELISA. Results from a representative experiment using T cell from a single donor are shown.

(B) Measurement of IFN-γ in supernatants from HeLa-L and HeLa-M in co-culture with γδ T cells. Either a concentration gradient of HMB-PP in co-culture (top panel) or a time-course of HMB-PP (at 10 nM) pre-treatment (bottom panel) was used to induce activation, before addition of T cells at E:T ratio of 10:1. After overnight incubation, culture supernatants were collected and analysed for IFN-γ by ELISA. Figure shows colour photographs of resulting ELISA plates. Conditions used for plasma-membrane profiling experiments of HMB-PP treated HeLa-M cells are highlighted (red boxes).

(C) T cell activation as detected by production of TNF-α from co-cultures of HeLa-L and HeLa-M cells pre-treated with HMB-PP (0-10 nM for 5 h) before addition of Vγ9/Vδ2 T cells. Graph shows results representative of triplicate experiments using T cells from different donors.

(D) A murine T cell hybridoma expressing human Vγ9/Vδ2 transgenes can be activated to produce IL2 by co-culture with HMB-PP loaded HeLa-L versus HeLa-M cells. Graphs show mean and sem from triplicate measurements.

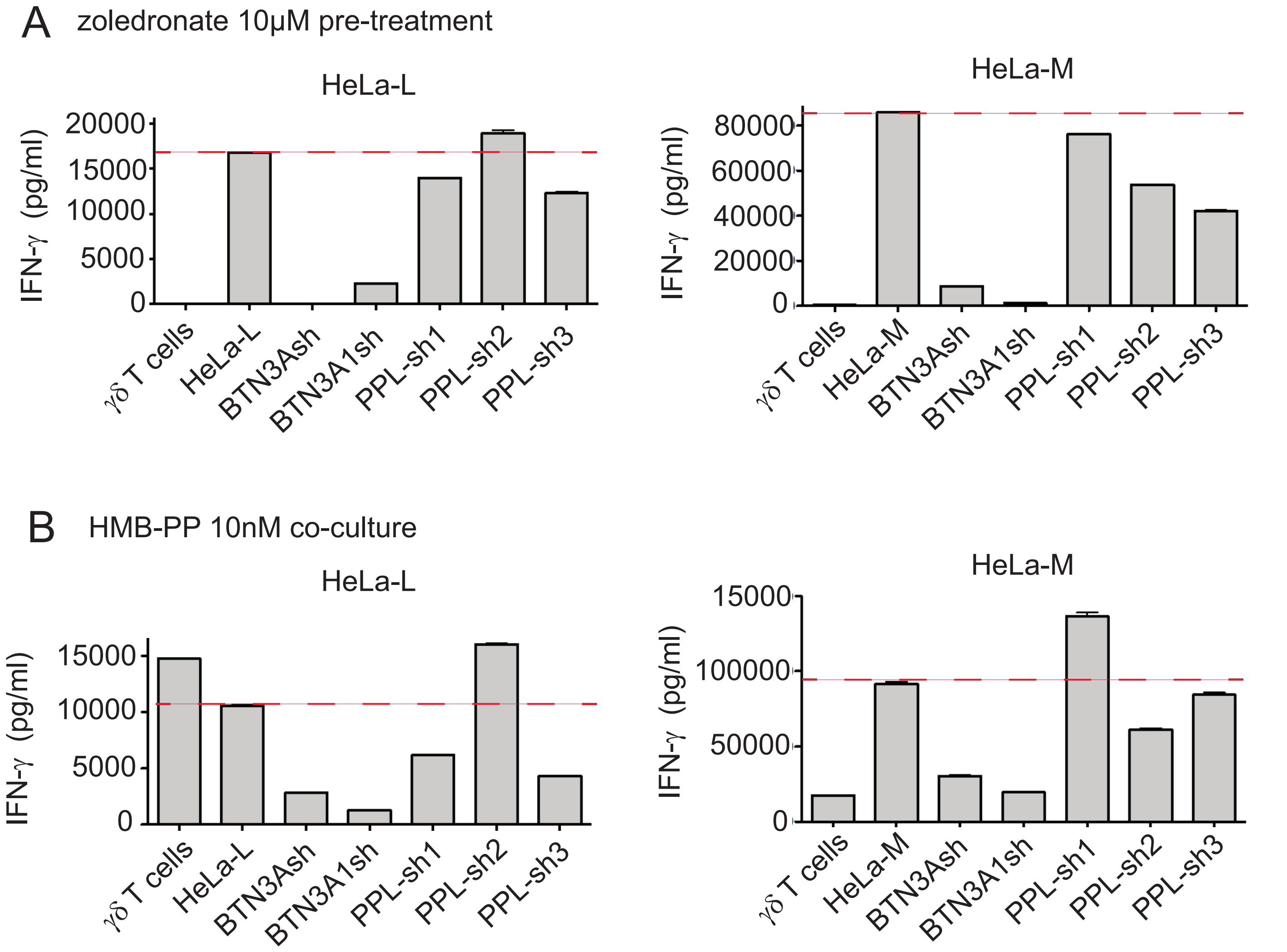

Supplementary Figure 2
(A) Analysis of PPL knockdown in HeLa-L and HeLa-M cells. Co-culture of γδ T cells with HeLa-L (left panel) and HeLa-M (right panel) wild-type cells, HeLa-M BTN3A (triple BTN3A knockdown by shRNA), HeLa-M BTN3A1 (BTN3A1 shRNA knockdown) and periplakin (PPL) knockdown lines PPLsh#1, PPLsh#2, and PPLsh#3, produced by expression of three separate targeting shRNA. T cell activation was induced using 10µM zoledronate and IFN-γ secretion into culture medium was detected by ELISA. An effector:target ratio of 10:1 was used. Data shown are representative of three independent experiments, with error bars (SEM).
(B) T cell activation by measurement of IFN-γ induced by 10 nM HMB-PP in co-culture with HeLa wild-type, BTN3A shRNA and PPL shRNA and γδ T cells as in (A).

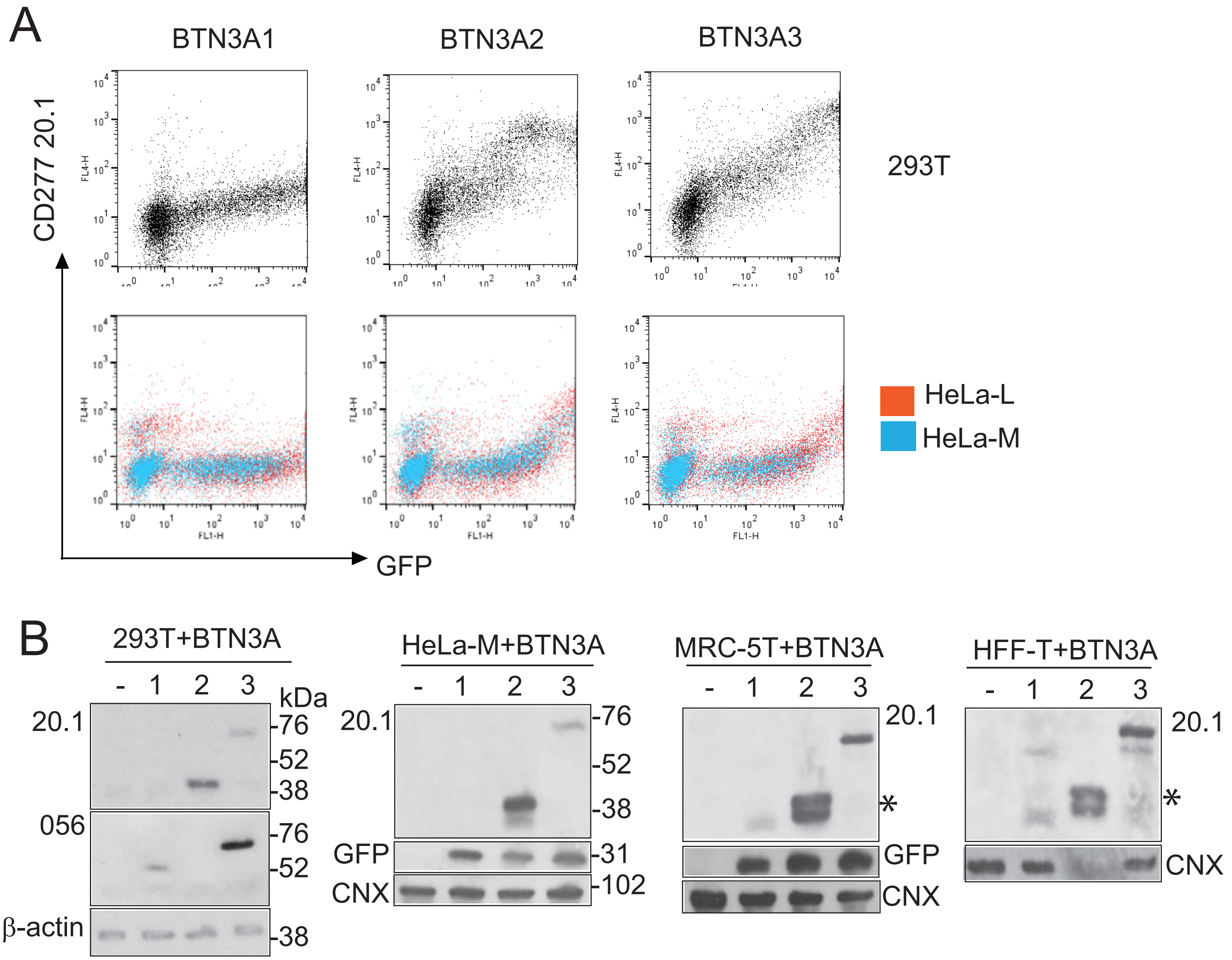

Supplementary Figure 3

(A) Transfection of DNA expression constructs for BTN3A1, BTN3A2 and BTN3A3 into 293T, HeLa-L and HeLa-M cells, analysed for surface expression of BTN3A using CD277 20.1 antibody. Results representative of triplicate experiments.
(B) Multiple cell lines were transfected with expression constructs for BTN3A1, BTN3A2 and BTN3A3 and analysed by Western blot using anti-BTN3A CD277 20.1 antibody. Anti-GFP and anti-calnexin (CNX) or anti-β-actin antibodies were used on duplicate blots as controls for expression of transgene and endogenous protein respectively. Isoform specific antibodies directed to B30.2 domains of BTN3A1 (056) and BTN3A3 (B6) were used to confirm expression in some experiments. Detection of CD277 20.1 and 056 reactive protein bands required extended exposure to X-ray film. Doublet bands of BTN3A2 protein detected by 20.1 antibody indicative of variation in post-translation modification are indicated (*).
